# Structural Angle and Power Images Reveal Interrelated Gray and White Matter Abnormalities in Schizophrenia

**DOI:** 10.1155/2012/735249

**Published:** 2011-10-10

**Authors:** Lai Xu, Tülay Adali, David Schretlen, Godfrey Pearlson, Vince D. Calhoun

**Affiliations:** ^1^The Mind Research Network, Albuquerque, NM 87106, USA; ^2^Department of ECE, University of New Mexico, Albuquerque, NM 87131, USA; ^3^Department of CSEE, University of Maryland Baltimore County, Baltimore, MD 21250, USA; ^4^Department of Psychiatry, Johns Hopkins University School of Medicine, Baltimore, MD 21287, USA; ^5^Olin Neuropsychiatry Research Center, Institute of Living, Hartford, CT 06106, USA; ^6^Department of Psychiatry, Yale University School of Medicine, New Haven, CT 06520, USA

## Abstract

We present a feature extraction method to emphasize the interrelationship between gray and white matter and identify tissue distribution abnormalities in schizophrenia. This approach utilizes novel features called structural phase and magnitude images. The phase image indicates the relative contribution of gray and white matter, and the magnitude image reflects the overall tissue concentration. Three different analyses are applied to the phase and magnitude images obtained from 120 healthy controls and 120 schizophrenia patients. First, a single-subject subtraction analysis is computed for an initial evaluation. Second, we analyze the extracted features using voxel based morphometry (VBM) to detect voxelwise group differences. Third, source based morphometry (SBM) analysis was used to determine abnormalities in structural networks that co-vary in a similar way. Six networks were identified showing significantly lower white-to-gray matter in schizophrenia, including thalamus, right precentral-postcentral, left pre/post-central, parietal, right cuneus-frontal, and left cuneus-frontal sources. Interestingly, some networks look similar to functional patterns, such as sensory-motor and vision. Our findings demonstrate that structural phase and magnitude images can naturally and efficiently summarize the associated relationship between gray and white matter. Our approach has wide applicability for studying tissue distribution differences in the healthy and diseased brain.

## 1. Introduction

Structural magnetic resonance imaging (sMRI) obtains high-resolution structural images that are useful for brain morphometry investigation. In sMRI images, two types of brain tissue, gray matter and white matters, are clearly perceptible and distinguishable. Usually, these two tissues are analyzed separately in studies of both healthy and diseased brain [1–3]. However, the relationship between gray and white matters is complicated. Gray matter is composed predominantly of cell bodies while white matter is composed mainly of axons connecting cell bodies; both are highly integrated within cerebral cortex and subcortical structures; spatial expansion of one can be associated with contraction of the other [4, 5]. Therefore, it is reasonable to expect that morphometric changes in one tissue may result in or be related to disturbance of the other. 

Several previous approaches have examined the relationship between gray and white matters. In voxel-based morphometry (VBM) studies, sMRI images were segmented first and the voxelwise correlation between regional cerebral gray and white matters was calculated [6, 7]; in region of interest (ROI) studies, gray and white matters were correlated with volumes in the rest of the cortex [8]. These correlation studies addressed the intricate relationship between gray and white matters and provided evidence of gray and white matter relative differences between diagnostic groups. One limitation of these approaches is that the correlations can only be calculated between individual voxels or between averages within prespecified regions. More complicated gray and white matter relationships can be studied by using univariate ANCOVA [9] or by using multivariate independent component analysis to identify linked gray and white matter networks [10]. In the current study, we propose a new approach to directly extract new features for gray and white matter fusion. The extracted angle and power features are sensitive to the gray and white matter interrelationship and can be used for single-subject diagnostic analysis or for group level analysis.

Schizophrenia affects multiple brain regions including both gray and white matters [11], it is likely that the inter-relationship between gray and white matters is affected in this mental illness. The disconnection model of schizophrenia [12] has led to increased focus on both gray and white matter analysis. Reviews of structural brain imaging in schizophrenia [11, 13] highlighted multiple regional abnormalities; reviews of white matter changes [14, 15] suggest that white matter disconnections are associated with the abnormalities, and reports of the corpus callosum and thalamus [16–18] identified subcortical regions whose abnormalities would likely reflect disturbances in circuits of multiple structural systems.

In this paper, our feature extraction method was applied to a large data set of healthy controls and schizophrenia patients, and the corresponding structural angle and power images were computed. As an initial evaluation, we performed a subtraction analysis between a single schizophrenia patient and a single healthy control. We then performed a univariate VBM analysis to detect the group level abnormalities in a voxelwise manner. Finally, an SBM analysis was used to detect structural networks covarying in a similar way which were related to the schizophrenia disturbances.

## 2. Methods

### 2.1. Subjects and Imaging Parameters

One hundred and twenty participants with schizophrenia (SZ) (mean age 42.1, SD 12.9, range 20–81, 51 females) and 120 matched healthy controls (mean age 42.7, SD 16.6, range 18–78, 65 females) were scanned at Johns Hopkins University. Exclusion criteria for all participants included a history of overt brain disease, mental retardation, head injury with loss of consciousness for greater than 60 minutes, or a diagnosis of substance abuse within the last year or lifetime substance dependence. Healthy participants were recruited using random digit dialing as part of Phase 1 of the Johns Hopkins aging, brain imaging, and cognition (ABC) study [19], a representative community sample. All healthy controls were screened to ensure they were free from current major depression, bipolar disorder, schizophrenia, and severe anxiety disorders using the schedule for clinical assessment in neuropsychiatry (SCAN) interview [20]. Patients met DSM-IV criteria for schizophrenia on the basis of the diagnostic interview for genetic studies (DIGSs) and review of the available medical records [21]. All patients with schizophrenia were stable and taking antipsychotic medications (precise medication information was not available for these data). These data were previously analyzed using SBM [22] and jSBM [10]. 

Whole brain sMRIs were obtained on a single 1.5*T* scanner (Signa; GE Medical Systems, Milwaukee, Wis). The whole brain was evaluated in the coronal plane using a spoiled GRASS 3D imaging sequence, with the following imaging parameters: 35 ms TR, 5 ms TE, 45° flip angle, 1 excitation, 1.5 mm slice thickness, 24 cm field of view, and a matrix size of 256 × 256.

### 2.2. Image Preprocessing

The images were preprocessed using the preprocessing steps typically applied for VBM [23, 24] and employed the Matlab program SPM5 (Statistical Parametric Mapping, Welcome Institute, London, UK). Images were normalized to the 152 average *T*_1_ Montreal Neurological Institute (MNI) templates, interpolated to voxel dimensions of 1.5 × 1.5 × 1.5 mm and segmented into gray matter, white matter, and cerebrospinal fluid (CSF) compartments. Registration, bias correction, and tissue classification were combined within one generative Gaussian mixture model which takes image intensity nonuniformities and tissue probability maps into consideration. The model parameter estimation involves alternating among classification, bias correction, and registration steps and aims to maximize the posterior solution of the three compartments. Then, the segmented gray matter and white matter images were smoothed separately with 12-mm full width at half-maximum (FWHM) Gaussian kernel. Each voxel in a smoothed image contains the averaged concentration of gray matter or white matter from around and within the selected voxel, a value ranging from 0 to 1. Next, we generated a mask using the smoothed gray and white matter images. The corresponding gray and white images were added together and then averaged. The averaged image was threshold at 0.1 and used to mask the smoothed gray or white matter images in order to exclude regions of very low concentrations (less than 0.1 of either gray or white matter). The masked gray and white matter images were used in the following steps. The size of each processed image was 121 × 145 × 121 voxels.

### 2.3. Gray and White Matter Fusion: Structural Angle and Power Images

**Figure 1:**
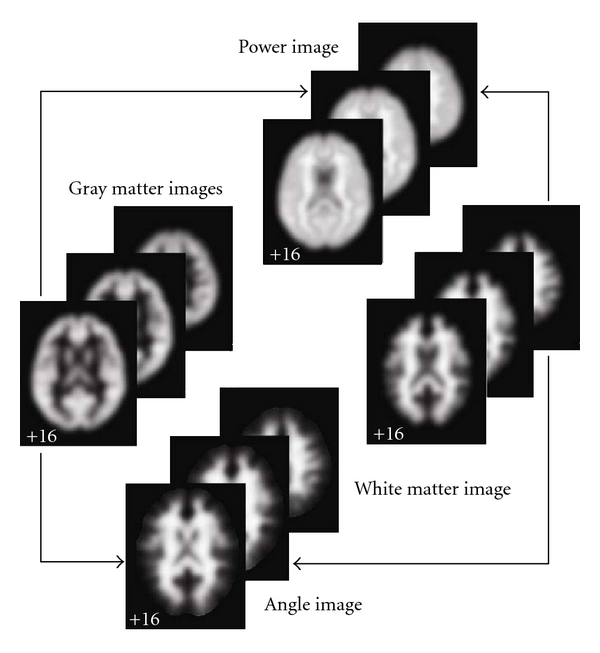
Structural angle and power image generation. Structural angle and power images are generated from the segmented gray and white matter images.

Structural angle and power images are then computed in order to combine/fuse gray and white matter tissue estimates. The overall scheme we use for image generation is represented in Figure 1. From one sMRI image, we obtain gray matter and white matter images. We assume the gray matter concentration within each voxel in the gray matter image is *g*_*i*_ and the white matter concentration within each voxel in the corresponding white matter image is *w*_*i*_, *i* = 1,…, 121 × 145 × 121. Next, we construct a complex variable *g*_*i*_ + *jw*_*i*_ for each and every voxel where *g*_*i*_ is the gray matter concentration and *w*_*i*_ is the white matter concentration. Thus, the phase/angle part of the complex variable is *φ*_*i*_(*g*_*i*_, *w*_*i*_) = arc tan⁡(*w*_i_/*g*_i_). The magnitude/power part of the complex variable is $M_{i}\left(g_{i},w_{i}\right)=\sqrt{g_{i}^{2}+w_{i}^{2}}$. The structural angle image is then constructed based on the angle value of each voxel, and the structural power image is constructed based on the power value of each voxel. 

Compared to approaches that work with the segmented gray and white matter images, the structural angle and power images we define emphasize more the relationship of gray and white matter distribution instead of focusing on the pure gray or white density. The angle image reflects the relative contributions of gray and white matter within each voxel and is proportional to the gray and white matter ratio changes. The power image is the mean power of the gray and white matter tissue concentrations and is proportional to overall tissue concentration. Thus, the angle and power images naturally and efficiently fuse gray and white matter together to emphasize the relationship between tissues and enable subsequent analysis without increasing computing complexity. Next, we apply three different methods, single-subject subtraction, VBM and SBM, to provide intuition and explain the utility of the structural angle and power images to study brain structure.

### 2.4. Single-Subject Subtraction Analysis of Structural Angle and Power Images

The structural angle and power features highlighted the interrelationship of gray and white matter for each subject. We performed a simple subtraction of randomly selected angle/power images between one healthy control and one patient to demonstrate the approach.

### 2.5. VBM Analysis of Structural Angle and Power Images

In order to detect statistically meaningful group differences, VBM was performed on the angle and power image set using SPM5. The 240 structural angle images and 240 structural power images were directly entered into a two sample *t*-test separately. The resulting angle/power *t*-maps highlight voxels that showed significant differences in angle/power between healthy controls and patients. The *t*-maps were then converted to angle/power *Z*-maps and thresholded at a value of |*Z*| > 3.0 for visualization.

### 2.6. SBM Analysis of Structural Angle and Power Images

We also performed an SBM analysis to identify structural networks showing group differences and common intersubject covariation. SBM was performed on the angle/power image set using the GIFT toolbox. SBM [22] is an approach that has been successfully applied to identify gray or white matter sources separately in sMRI images. A “source” is a network comprising several regions that together exhibit intersubject covariance and show group differences. Compared to VBM, SBM is a multivariate data-driven method taking cross-voxel information into account, which results in group differences that are represented by maximally independent sources not voxels as in VBM. Our previous work [22] has shown the effectiveness of applying SBM for network detection in segmented gray matter images. Here we applied SBM to structural angle and power images to detect networks of relative gray and white matter changes. This SBM approach consists of three steps: independent component analysis (ICA), statistical analysis, and visualization.

**Figure 2:**
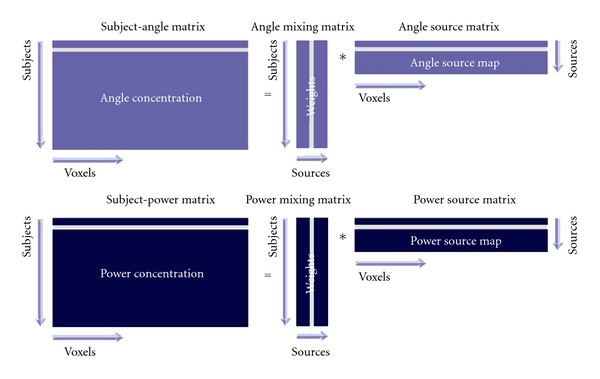
Independent component analysis on angle and power images. Angle or power images are stacked into one subject-angle/power matrix. ICA is then used to decompose this matrix into a mixing matrix and a source matrix. The mixing matrix is used for statistical analysis and the source matrix is used for sources visualization in the following steps.

First, ICA is performed on the angle images and power images separately (see Figure 2). We take the angle images as our example. Each angle image is converted to a one-dimensional vector. The 120 angle image vectors of healthy controls and 120 angle image vectors of patients are then arrayed into one 240 row subject-by-angle matrix. Akaike's information criterion (AIC), modified to improve the estimation performance for medical images [25], was applied to the matrix in order to estimate the number of sources *k*. Next, the subject-by-angle matrix was decomposed into a subject × source *angle mixing matrix* and source × angle *angle source matrix* using spatial ICA [26]. The angle mixing matrix expresses the relationship between 240 subjects and *k*-angle sources. The rows of the matrix are scores that indicate to what degree each of the *k*-angle sources contribute to a given subject. The columns of the matrix indicate how one angle source contributes to the 240 subjects. In contrast, the angle source matrix expresses the relationship between the *k*-angle sources and the voxels within the brain. The rows of the matrix indicate how one angle source contributes to different brain voxels and the columns of the matrix are scores that indicate how one voxel contributes to each of the angle sources. The same process was applied to the 240 power images to determine the *power mixing matrix* and *power source matrix*.

Then statistical analysis was performed on the angle mixing matrix and power mixing matrix separately. Since every column of the mixing matrix contains the loading parameters expressing the contribution of every source for the 240 subjects, a two-sample *t*-test can be used on each column of the mixing matrix to test which source shows a significant control versus schizophrenia difference. A corrected threshold of *P* < 0.05, controlling for the false discovery rate (FDR), was used to identify the most significant sources [27]. The effects of age and gender on the significant sources were also determined. We regressed the columns of the mixing matrix on these variables using a threshold of *P* < 0.05 to determine sources that were significantly correlated with them. In order to verify that the group differences in the significant sources were still present after removing the effect of age and gender, we computed a two-sample *t*-test on the residual of the regression and tested the difference between controls and patients.

Finally, the source matrix was used for visualization. The angle source maps were obtained from the angle source matrix and power source maps were obtained from the power source matrix. Each row of the source matrix was scaled to unit standard deviation, then reshaped separately into one 3D image (source map). The significant source maps were then superimposed on the MNI-normalized template brain and thresholded at |*Z*| > 3.0. Regions within the most significant sources were labeled by transforming from the MNI coordinate system to the coordinates of the standard space of Talairach and Tournoux [28] using a Matlab conversion program (http://imaging.mrc-cbu.cam.ac.uk/downloads/MNI2tal/; MRC Cognition and Brain Sciences Unit, Cambridge, England). Once converted, the Talairach coordinates were entered into the Talairach daemon [29] and summarized. In addition, white matter regions within significant sources were thresholded at |*Z*| > 3.0 and specifically labeled using the ICBM DTI-81 atlas [30].

### 2.7. Simulations

**Figure 3:**
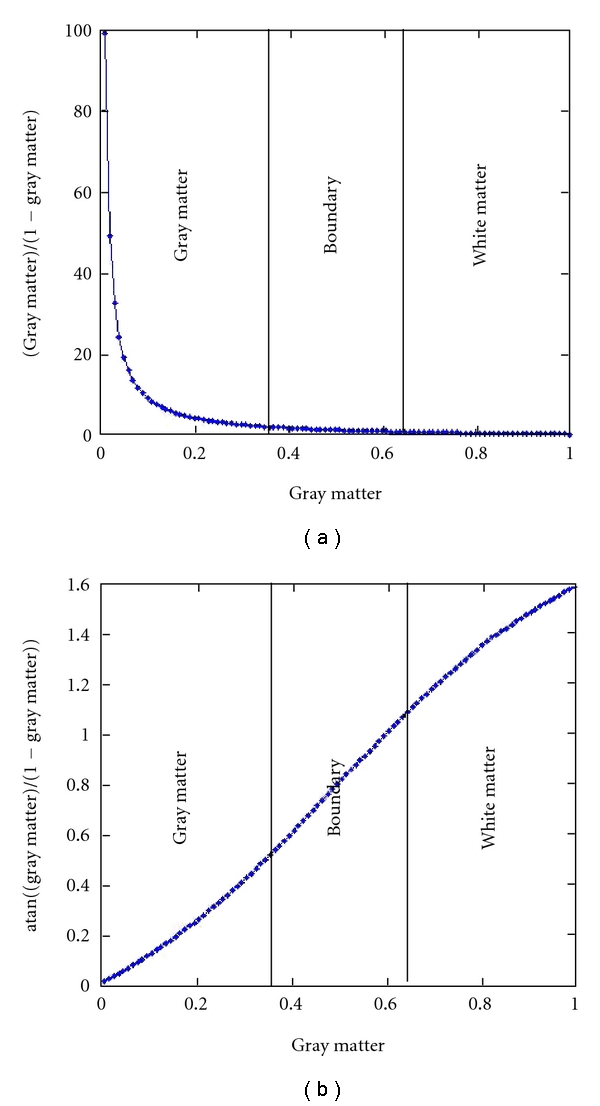
Evaluation of atan function.

Figure 3(a) presents a simple plot of gray matter versus the ratio of white matter/gray matter (assuming for simplicity that gray matter = 1 − white matter). Approximate cases with mostly gray matter, mostly white matter, and in between (boundary regions) are denoted on the plots as well. It is clear this is a highly nonlinear relationship with the function going to infinity as gray matter approaches zero.  Figure 3(b) shows that gray matter versus the structural angle is much more linear, with some smaller nonlinearities as gray matter or white matter goes to zero. This squashing of the instability near zero is a very useful property of the atan function. Also note that the slope is steeper than that for gray matter. As we will see in the next simulation, the structural angle also provides increased sensitivity to group differences compared to using gray matter alone.

**Figure 4:**
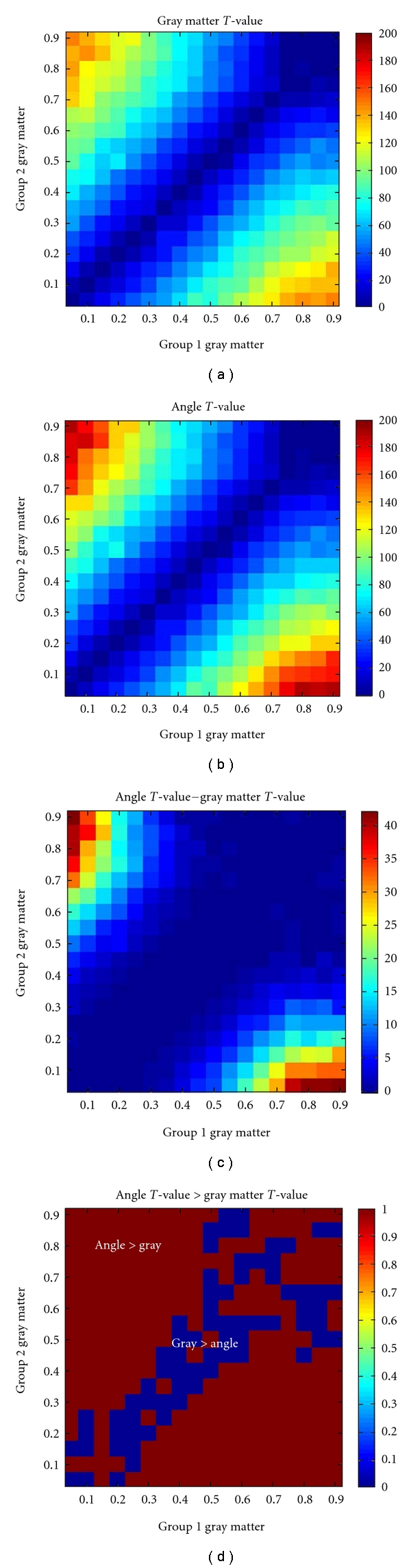
Simulation results. Group differences in gray matter or angle for a range of gray matter values from 5% to 100%. (a, b) show group difference *T*-values for gray matter and angle, respectively. The patterns are quite similar, although the angle shows generally larger *T*-values (see colorbar scales). (c) shows the difference in *T*-values. (d) shows that for most combinations of gray matter, the *T*-values for angle are larger than those for gray matter (red regions in the image) especially in regions where one group has larger gray matter values and the other has smaller gray matter values.

In order to better understand the added value of using the angle measure, we performed a simulation of a single voxel in a group of 100 subjects in group 1 and 100 subjects in group 2. We generated data for a range of gray matter values from 5% to 90% in a given voxel for each group. For each gray matter, setting a small amount of random (uniform) noise was added to each voxel. White matter voxels were then calculated assuming that white matter = 1 − gray matter. Once this data was generated, we computed the two-sample *T*-values for group 1 versus group 2 for either the gray matter values along (analogous to standard VBM) or for the angle arc tan⁡(*w*_*m*_/*g*_*m*_). Results are presented in Figure 4. In general, the pattern of the *T*-values is quite similar for both gray matter and angle (*T*-values and log of absolute *T*-values are shown). Unsurprisingly, the largest *T*-values for both measures occur when one group have large gray matter values and the other group has small gray matter values. The difference in these *T*-values tells us where the sensitivity is greater for either gray matter or angle.  Figure 4(d) (red regions) show where the angle value is greater than the gray matter value. In general, the angle measure is providing more sensitivity to the group differences than gray matter alone, especially where one group has larger gray matter values and the other group has smaller gray matter values. This includes, but is not limited to, regions where boundaries between gray matter and white matter are shifted in the two groups (e.g., where the gray matter in one group drops off faster than that in the other group). In contrast, the power images have the greatest sensitivity to group differences where the gray matter value of one group is more similar to that of the other group and when both groups have larger gray matter values. 

## 3. Results

We propose structural angle and power as two new features describing the interrelationship of gray and white matters. We show the results of the three different analyses performed on the structural angle and power images extracted from the sMRI images of healthy controls and schizophrenia patients below. We also show an application of these features to study schizophrenia.

### 3.1. Results of Single-Subject Subtraction: Single-Subject Abnormality

**Figure 5:**
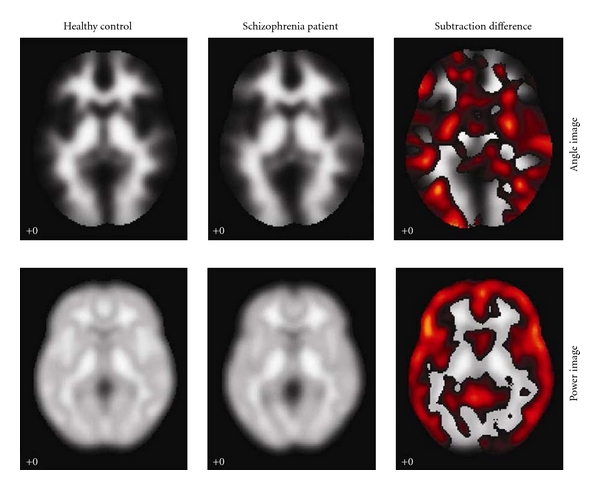
Subject differences in angle and power. The upper row consists of angle images and the bottom consists of power images. The first column is the images from healthy control; the second is the images from the schizophrenia patient; the third is the subtraction showing the subject differences.

By simply subtracting the structural angle and power images between subjects, we highlighted the regions showing subject differences of overall gray and white matter distribution. As shown in Figure 5, the upper row consists of angle images and the bottom consists of power images. The first column shows the images from one healthy adult. The second shows images from one patient with schizophrenia. The third depicts subtraction-related differences in the angle and power images. Compared to the healthy adult, the patient with schizophrenia showed higher angle values in middle temporal and frontal gyri, precuneus and cuneus, cingulum, the body and splenium of corpus callosum, and lower power values in superior and inferior frontal gyri, superior temporal gyrus, and fornix.

### 3.2. Results of VBM Analysis: Group Level Differences

**Figure 6:**
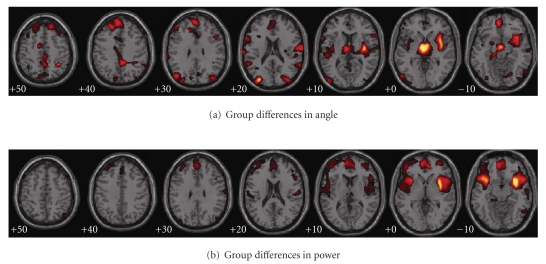
Group differences in angle and power detected by VBM. The regions were thresholded at |*Z*| > 3.0.

By applying VBM on the structural angle and power images, the statistical *Z*-maps (see Figure 6) reflecting the group differences of relative gray and white matter between controls and patients were obtained. The Talairach coordinates for the maps are listed in Table 1. The white matter determined by ICBM DTI-81 atlas is listed in Table 2.

The angle map reveals significantly higher white-to-gray ratio for patients with schizophrenia in thalamus, internal capsule, insula, cuneus and precuneus, superior and middle frontal gyri, inferior frontooccipital fasciculus, and uncinate fasciculus. The power map shows the most significant average concentration differences between the diagnostic groups in bilateral superior temporal gyrus, medial and superior frontal gyri, claustrum, external capsule, cingulum, inferior frontooccipital fasciculus and uncinate fasciculus. The temporal regions are notably constrained to the superior temporal gyrus and its medial counterparts, the transverse temporal gyrus and insula, suggesting a clear distinction between these structures and the rest of the temporal lobe.

### 3.3. Results of SBM Analysis: Network Disturbances

**Figure 7:**
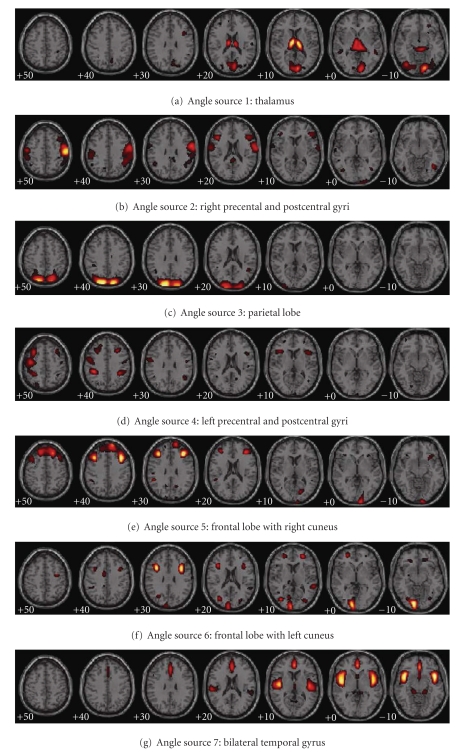
Angle and power networks detected by SBM. Six angle networks showing significant gray-to-white matter ratio abnormalities and one power network, showing significant average concentration reduction, were shown. The regions were thresholded at |*Z*| > 3.0.

Through SBM analysis, sources that are formed by networking regions showing the same intersubject covariance can be detected. The number of angle sources was estimated to be 25, and the number of power sources to be 37 using the modified AIC approach. Eight angle sources and three power sources were identified as having loading parameters that significantly differed between controls and patients. On visual inspection of the source maps, two angle sources and two power sources appeared to be obvious artifacts showing sharp edges near the brain boundary or appearing within CSF regions. Within the remaining six angle sources and one power source, the loading parameters of patients in the mixing matrix were all lower than those of controls. Each of the identified sources includes regions reflecting group differences in angle/power covariation among subjects (see Figure 7). The Talairach coordinates for the sources are listed in Table 3. The white matter determined by ICBM DTI-81 atlas is listed in Table 4. The analysis of age and gender effects on these sources was also given. For convenience, the sources are listed by a summary of their anatomical regions and represented in order of increasing *P* values (decreasing significance). Note that since each source represents a set of regions, the short anatomic label does not fully describe them.

**Angle Source 1: Thalamus.**

The most significant angle difference between controls and schizophrenia was in angle source 1. Within this source, the angle value was larger (e.g., the white/gray ratio was higher) for patients than controls in thalamus, lingual gyrus, cuneus, precuneus, inferior occipital gyrus, retrolenticular part of internal capsule, fornix, and cingulum.

**Angle Source 2: Right Precentral and Postcentral Gyri.**

This source presented the second angle significant difference between healthy controls and patients with controls having lower angle values (e.g., less gray and more white matter) in postcentral gyrus, precentral gyrus, inferior and middle frontal gyri. The distribution lays particular emphasis on the right hemisphere with much larger volume and maximum value.

**Angle Source 3: Parietal Lobe.**

This source included regions of cuneus, precuneus, and superior parietal lobule with lower gray-to-white matter ratios in patients than controls.

**Angle Source 4: Left Precentral and Postcentral Gyri.**

This source showed significant difference between controls and patients, with controls having less gray and relatively more white matter partition in postcentral gyrus, precentral gyrus, superior and middle frontal gyri. The regions of both angle sources 2 and 4 mainly involve prefrontal and postfrontal gyri, with angle source 4 distributing more to the left hemisphere and angle source 2 more to the right hemisphere.

**Angle Source 5: Right Cuneus with Frontal Lobe.**

More gray and less white matter partitions in healthy controls than patients were found in this source, which included middle and superior frontal gyri and the right cuneus.

**Angle Source 6: Left Cuneus with Frontal Lobe.**

This also showed a significant angle difference between controls and patients, with controls having more gray matter partition in middle frontal gyrus, left lingual gyrus, and left cuneus. Both angle source 5 and 6 occur mainly in cuneus and middle frontal gyrus, with angle sources 5 emphasizing the right and source 6 emphasizing the left cuneus.

**Power Source: Bilateral Temporal Gyrus.**

The most significant source showing average concentration differences between the diagnostic groups was found in bilateral superior temporal gyri, insula, anterior cingulate, medial and inferior frontal gyri, cingulum, and uncinate fasciculus. Healthy controls consistently showed more average gray and white matter concentration than patients.

**Age and Gender Effects.**

**Figure 8:**
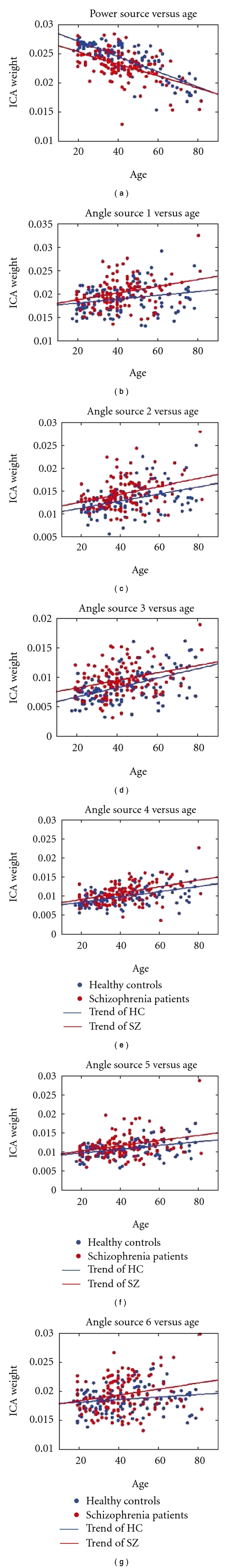
The correlation plots between age and ICA weights for angle and power sources. Red dots: correlation for the patients; blue dots: correlation for the controls; red line: trend for red dots; blue line: trend for blue dots.

There was no significant effect of gender on any source. There was a significant effect of age on all sources at *P* < 0.005. The correlation plots of age versus ICA weights for the sources are presented in Figure 8. The ICA weight increases as age increases of all the angle sources according to the linear trend. For angle sources 1, 4, 5, and 6, the intercept value of controls is higher than that of patients, and the slope values of controls and patients are nearly identical. For angle source 2, the intercept value of controls is higher than that of patients, and the slope value of controls and patients are nearly the same. For angle 3, the intercept value of controls is higher than that of patients, and the slope value of controls is slightly lower than that of patients. For the power source, the ICA weight decreases as age increases. According to the linear trend, the intercept value of the controls is higher than that of patients and the negative slope value of the controls is slightly lower than that of the patients. After removing effects of age and gender, the group differences in the sources remained significant.

## 4. Discussion

To our knowledge, this is the first study to extract interrelated features of gray and white matter for brain structural analysis. Three different analyses were applied to the angle and power images. A single-subject subtraction highlighted the interrelated tissue distribution differences at the individual subject level. A univariate VBM analysis detected group level differences between healthy controls and schizophrenias, which offered statistical maps of fused gray and white matter abnormalities. A multivariate SBM analysis further filtered the noise and determined several networks showing group differences. We also evaluated age and gender effects on the networks.

### 4.1. Tissue Distribution Showing Subject Differences

The angle and power images emphasize the interrelated gray and white matter concentration. The angle image reflects the gray-to-white matter ratio and is sensitive to small changes in regions where gray matter is increasing and white matter is decreasing (or changing little), or vice versa. The power image indicates overall tissue concentration and highlights tissue presence in each voxel, especially in regions where both gray and white matter concentrations are low. Subtraction of angle/power images between healthy controls and schizophrenia patients shows tissue distribution differences between two subjects. Results suggest richer information showing that such differences can be captured by the angle and power images. The angle value differentiation showed a smaller gray-to-white matter in patients versus controls in a wide range of areas of both gray and white matters. Power images also revealed patient/control differences in the superior temporal gyrus, suggesting that both gray and white matters are lower in this region in patients.

One of the key advantages of our approach is the ability to evaluate changes in both gray and white matters through the structural angle and power images. This provides a complementary approach to methods which work only with gray matters images. In addition, the simulation we performed suggests increased sensitivity to group changes over an approach which uses only the gray matter images. Comparison with a previous paper in which we analyzed the gray matter images with both VBM and SBM highlights the complementary nature of our proposed approach. In [22], we identified multiple SBM sources which showed group differences in patients and controls. Five sources were identified as summarized in Table 5. Though an exact match was not possible, since there are some differences in the regions included in the sources, an approximate match is provided in the table. We can see from this that all but one of the sources were identified in the angle and power analyses we performed in the current paper. The basal ganglia region was identified in the gray matter analysis but not the angle or power analysis. As discussed in [22], the SBM approach has some important advantages over voxel-based approaches since it groups regions which have common intersubject covariation together. In addition, noise sources are typically separated into separate sources, thus providing a sort of spatial filter to clean up the remaining sources. In this paper, we proposed a transformation of the gray matter data into structural angle and power images. This approach has some advantages and in particular appears to be more sensitive to subtle group differences, especially where one group has high gray matter values and the other group has low gray matter values. This also includes, but is not limited to, regions where boundaries between gray matter and white matter are shifted in the two groups (e.g., where the gray matter in one group drops off faster than that in the other group). However, our approach should be seen as a complementary approach; not meant to replace analyses of gray or white matter separately. Next, we summarize and discuss the findings in the current analysis in more detail.

### 4.2. Tissue Distribution Showing Group Differences

The VBM analysis identifies group level differences of tissue distribution by voxel-by-voxel comparison. Results indicated that white matter concentration was higher and gray matter concentration was lower in the thalamus in schizophrenia. This is consistent with previous work showing thalamic gray matter reductions [17]. Changes in the insula, which received projects from the thalamus, is also in agreement with a previous report [31]. The findings of greater white-to-gray matter in superior and middle frontal gyri was consistent with the gray matter reduction in these regions [11]. The disruption of uncinate and inferior frontal-occipital fasciculi is consistent with previous reports [32, 33] as these association fibers project to the smaller cortical regions. In addition, the results also suggest a disturbance in cuneus and precuneus underlying the disease. We also showed changes in the internal capsule that also receives thalamic projections.

Regions showing significant differences in gray and white matter average concentration also revealed a large continuous region of temporal lobe that included the bilateral superior temporal gyrus, planum temporale, transverse temporal gyrus, and insula, but little of middle or inferior temporal regions, consistent with previous reports of selective gray matter reductions in the temporal gyrus [13, 34]. Also the concentration disruption in medial and superior frontal gyri agrees with previous findings of gray matter reduction in sMRI studies [11]. Our findings also suggest that the claustrum and external capsule should be further studied as they play an important role in cortico-cortical connections.

### 4.3. Tissue Distribution Showing Network Abnormalities

The SBM analysis enables evaluation of maximally independent features which also show similar intersubject covariation which differ in degree between patients and controls. Consistent with our simulation, the structural angle feature identified the most sources showing group differences. The power angle identified an important previously identified network in which both groups have larger gray matter values. We now discuss the difference sources in more detail.

Angle source 1 suggested the abnormality of a thalamic structural network in schizophrenia. The higher thalamic white-to-gray ratio detected by SBM confirmed the evidence detected by the VBM analysis. The smaller cuneus and lingual gyrus angle agree with the lesser occipital lobe gray matter reported by others [35] and abnormalities in fornix and cingulum are also consistent with previous studies [36, 37]. Our findings suggested that the posterior thalamic projection which penetrates the retrolenticular part of internal capsule and connects to the occipital lobe through cingulum was abnormal in patients. We also suggested that the anterior thalamic projection might be affected by the fornix disruption.

Angle sources 2 and 4 were two networks comprising tissue distribution disturbances in right and left hemispheres sensory-motor cortex, respectively. The smaller gray matter partition of precentral and postcentral gyri in patients is consistent with previous studies [38, 39]. Since postcentral gyrus contains the main sensory receptive area of primary somatosensory cortex and the dorsal part of the precentral gyrus is the location of primary motor cortex, these two angle sources looked similar to a functional sensory motor pattern, one containing the left hemisphere and the other the right. Our findings suggest that these two structural sources in precentral and postcentral gyri might underlie sensory-motor disturbances in schizophrenia and that structural information associated with functional areas might be identified by these tissue distributions.

Angle source 3 was mainly located in parietal lobe. Our finding of less gray-to-white matter ratio in parietal cortex is consistent with a previous report of more regional white matter and less gray matter in schizophrenia [9]. Since cuneus, precuneus, and parietal lobule are all involved in basic visual processing [40], the structural distribution abnormality might be related to the observed disturbances in the visual stream.

Angle sources 5 and 6 were two networks focusing on frontal lobe and cuneus. The lower gray-to-white matter ratio of patients in middle frontal gyrus and cuneus agrees with previous reports [32, 41, 42]. Our findings suggest that there might be connectivity between cuneus and frontal cortex, disturbances reflecting an abnormal working memory network.

The power source showed regions consistently identified as disrupted in schizophrenia. Since the unicinate fasciculus connects the frontal and temporal lobes and the cingulum bundle collects projections from the nearby cingulate gyrus and extends into the temporal lobe, this circuit can be considered a local area network describing frontal-temporal connectivity. Our findings agree with previous studies of frontotemporal connections [43, 44], providing supportive evidence for the disconnection hypothesis of schizophrenia.

All sources were maximally spatially independent and each revealed one network that differed significantly in schizophrenia versus controls. By examining the Talairach table and the figure of sources, some regions were shared by several sources. These overlapping regions corresponded to different structural connectivities, with patterns suggesting disruptions in higher cortical functions that appeared to be most disturbed in schizophrenia patients. For example, the lingual gyrus was shared by angle sources 1 and 6, which was part of the disturbance in both the thalamic network and cuneus network; the cingulum appears in angle source 1 and power source, which was frequently found to be structurally or functionally altered in individuals with schizophrenia [36, 45]; the middle and medial frontal gyri were in angle sources 2, 4, 5, and 6, which indicated the multifunctional roles of prefrontal cortex; the precuneus was observed in all of the angle sources, consistent with its participation in multiple functions [46] and it was likely that this region may serve as a hub of multiple naturally grouped networks.

Subject-specific loading parameters for all sources were significantly correlated with age in this cross-sectional study. For angle source 1, 4, 5, and 6, the intercept suggested that the white-to-gray matter ratios of patients and controls were similar at younger ages. However, in patients this ratio increased faster than controls with increasing age. At older ages, the gray matter partition in patients was smaller than in controls, and the white matter partition increases more in patients than in controls. For angle source 2, the white-to-gray matter ratio of patients was larger than that of controls and increases with age. For angle source 3, the intercept and slope suggested that the white-to-gray matter ratio of patients was larger than of controls at earlier ages, however, it declined faster than controls with increasing age. By age 75, the ratio reached a similar size for both patients and controls. For the power source, the average concentration in patients was less than that in controls at earlier ages and continued to decline with increasing age. At older ages, the concentration in patients and controls reached the same level.

## 5. Conclusion

In this paper, we demonstrate an approach to extract features by combining gray and white matter information in two different ways. The angle image reflects the partitions of gray and white matter within each voxel and the power image indicates the average tissue concentration. Both of them naturally capture interrelated changes in tissue distribution and are sensitive to the small changes in regions where gray and white matter concentrations are low. Three different analyses, single-subject subtraction, VBMs and SBMs were applied to the angle and power images separately to explain the utilization of the structural angle and power images in schizophrenia and to evaluate the efficiency of the features for interrelated gray and white matter fusion. These initial experiences with structural angle and power images revealed several interesting findings in schizophrenia that were not identified by standard, separate gray or white matter analyses and demonstrate the usefulness of angle and power joint gray and white matter assessment.

## Figures and Tables

**Table 1:** Talairach labels for regions detected by VBM. Voxels above a threshold of |*Z*| > 3.0 were converted from Montreal Neurological Institute (MNI) coordinates to Talairach coordinates and entered into a database to assign anatomic labels for the left (L) and right (R) hemispheres. The concentration of voxels in each area is provided in cubic centimeters (cc). The areas with volume above 1.0 are listed. Within each area, the maximum *Z* value and its coordinate are provided.

Angle	Brodmann area	L/R volume (cc)	L/R random effects: max *Z*(*x*, *y*, *z*)
Thalamus		6.0/6.7	6.3(1, −13,2)/6.5(−3, −9, −3)
Insula	13, 47, 41	6.9/2.4	5.9(−1, −7,2)/4.5(3, −3, −7)
Middle, superior and inferior occipital gyri	19, 37, 18	8.2/11.3	4.6(−31,4, −17)/5.7(1, −3, −2)
Anterior cingulate	25, 32, 24, 10	1.9/2.8	5.7(1, −15, −3)/4.0(34,5, −18)
Inferior, superior, medial, and middle frontal gyri	47, 13, 44, 45, 11, 46, 9, 10, 6, 8	32.5/26.9	5.4(7, −6, −5)/5.2(−1, −16,6)
Claustrum		4.8/1.5	5.2(3, −3,3)/4.2(−39, −17,12)
Inferior parietal lobule	40, 39	4.5/2.6	5.0(−7, −43,42)/4.1(−61,18,9)
Cingulate gyrus	31, 24, 32	4.5/6.0	5.0(−15, −76, −14)/4.7(−34,13,2)
Parahippocampal gyrus	34, Amygdala, 27, 36, 28, 35, 19	1.9/2.8	5.0(10, −9,5)/3.9(−27, −15,12)
Lentiform nucleus		3.7/1.7	4.9(−30,11, −18)/4.7(3,51, −9)
Postcentral gyrus	43, 40, 2, 1, 3, 7, 6, 13, 4, 44	6.2/5.0	4.8(−6, −13, −6)/4.5(45, −73,33)
Cuneus and precuneus	19, 18, 17, 7, 39, 31	9.2/9.1	4.7(9, −4,42)/4.7(−27, −75, −15)
Angular gyrus	39	1.5/1.1	4.2(−13, −84, −25)/4.7(7,0, 0)
Supramarginal gyrus	40	2.2/1.3	4.6(−34,14, −6)/3.6(−4, −25,3)
Superior, inferior and middle temporal gyri	38, 42, 22, 41, 13, 39, 21,19, 20, 37	13.4/12.9	4.6(−1,50,29)/4.3(−22,46,45)
Inferior semilunar lobule		9.1/2.8	4.5(−9, −80, −24)/3.8(−1, −55,8)
Cerebellar tonsil		11.4/1.1	4.5(−59, −69, −2)/3.5(−7, −10,10)
Paracentral lobule	31, 6, 5	2.2/0.6	4.3(30, −75, −15)/3.3(9, −66,46)
Cerebellar vermis		19.5/7.3	5.3(1, −19,2)/4.3(50, −77,29)
Lingual gyrus	18, 19	1.9/0.2	4.0(−37, −79,27)/3.5(67, −44,20)
Fusiform gyrus	37, 18, 19, 20	3.2/1.5	3.6(39, −7, −19)3.6(−53, −60, −10)

Power	Brodmann area	L/R volume (cc)	L/R random effects: max *Z*(*x*, *y*, *z*)

Claustrum		3.2/3.0	5.8(−34,5, −9)/4.5(−45,16, −10)
Inferior, superior, medial and middle Frontal gyri	13, 47, 11, 10, 45, 46, 47, 44, 9, 25, 6, 8	38/59.3	5.6(−34,13, −3)/4.8(−40,10, −7)
Insula	13, 40	5.2/4.8	5.6(−36,1, −3)/5.1(−30,8, −12)
Superior temporal gyrus	38, 22, 41, 13	14.3/16.6	5.0(−37, −3,2)/4.3(−45, −15, −41)
Inferior semilunar lobule		9.1/3.5	4.6(−52,14, −7)/3.8(−34,54, −3)
Parahippocampal gyrus	34, Amygdala, 36, 35, 27, 30	0.9/1.7	4.5(30,29, −23)/4.1(6,54,6)
Middle and inferior temporal gyri	21, 38, 39, 37, 19, 20, 25	6.7/5.0	4.4(−25,14, −15)/3.7(−22, −79, −33)
Cerebellar tonsil		2.4/1.1	4.2(24,28, −24)/3.4(−3,54,22)
Anterior cingulate	32, 25	1.9/1.9	3.6(3,46, −6)/3.9(−22, −59, −46)
Cerebellar vermis		6.4/4.9	4.3(−36,2, 5)/4.1(−49,9, −2)
Precentral and postcentral gyri	3, 7, 43, 40, 1, 44, 6, 13	6.9/5.4	3.7(−33, −72, −39)/3.6(−6,44,1)

**Table 2:** White matter labels for regions detected by VBM. Voxels above a threshold of |*Z*| > 3.0 were converted from Montreal Neurological Institute (MNI) coordinates to the ICBM DTI-81 coordinates and entered into a database to assign anatomic labels. The volume of significant white matter voxels within each fiber tract area is provided in cubic centimeters (cc). The areas with volume above 0.1 are listed. The percentage of the fiber tract containing significant white matter voxels is also provided. Within each fiber tract, the maximum *Z* value and its coordinate are provided.

Angle	L/R volume (cc)	L/R percentage (%)	L/R max *Z*(*x*, *y*, *z*)
Anterior limb of internal capsule	0.07/0.51	2.14/22.69	4.27(9,0, 2)/5.53(−9, −6, −3)
Posterior limb of internal capsule	0.63/0.09	16.61/2.94	6.17(8, −5,0)/4.52(−11,0, 2)
Retrolenticular part of internal capsule	0.25/1.40	10.05/36.40	3.87(−29, −20,12)/5.42(−9, −5,0)
Posterior corona radiata	0.16/na	4.23/na	3.72 (−18, −45,41)/na
External capsule	2.36/0.31	66.20/8.70	5.50 (−36, −9,6)/3.41 (32,8, 3)
Cingulum (cingulate gyrus)	0.26/0.23	9.74/9.87	3.41 (−8,20,30)/3.80 (6, −11,42)
Cingulum (hippocampus)	na/0.35	na/27.81	na/3.92(20, −29, −8)
Inferior frontooccipital fasciculus	1.37/0.03	72.42/1.41	5.00(−36, 3, −3)/3.09(29, 6, −3)
Uncinate fasciculus	0.37/0.06	100/16.8	4.46(−39,0, −15)/3.60(35, 3, −20)

Power	L/R volume (cc)	L/R percentage (%)	L/R max *Z*(*x*, *y*, *z*)

External capsule	0.88/0.36	10.42/24.72	5.69(−35,8, −2)/4.28(32,11, −2)
Cingulum (hippocampus)	na/0.13	na/10.96	na/3.60(23, −30, −12)
Inferior frontooccipital fasciculus	1.22/0.55	0.6478/28.87	5.99(−35,8, −8)/4.26(32,11, −3)
Uncinate fasciculus	0.31/0.35	83.64/92.92	5.42(−35,3, −11)/4.12(33,6, −11)

**Table 3:** Talairach labels for networks detected by SBM .Voxel above a threshold of |*Z*| > 3.0 were converted from Montreal Neurological Institute (MNI) coordinates to Talairach coordinates and entered into a database to assign anatomic labels for the left (L) and right (R) hemispheres. The concentration of voxels in each area is provided in cubic centimeters (cc). The areas with volume above 1.0 are listed. Within each area, the maximum *Z* value and its coordinate are provided.

	Brodmann area	L/R volume (cc)	L/R random effects: max *Z*(*x*, *y*, *z*)
*Angle source 1*:			
Thalamus		13.2/12.5	16.7(−10, −13,12)/15.7(10, −15,12)
Lingual gyrus	18, 17, 19	3.5/11.4	5.9(−16, −80, −2)/11.2(13, −83, −3)
Cuneus	18, 17, 30, 19, 7, 23	8.6/11.7	7.4(0, −84,17)/10.4(7, −85,11)
Inferior and middle occipital gyri	17, 19, 18, 37	8.0/5.4	7.4(−39, −73, −5)/9.3(13, −88, −7)
Culmen		1.3/1.7	7.8(−3, −32, −5)/8.2(3, −34, −5)
Precuneus	31, 7, 19	1.7/4.5	5.8(−9, −72,23)/7.4(6, −73,22)
Caudate		1.3/0.4	6.6(−7, −2,15)/4.7(18, −24,18)
Superior, Inferior and middle frontal gyri	6, 47, 11, 9, 10	2.8/7.3	6.3(−19, −11,72)/5.4(25,21, −16)
Parahippocampal gyrus	27, 35, 30, 36, 28	0.9/2.2	4.8(−15, −29, −6)/6.0(12, −34, −1)
Middle temporal gyrus	22, 21, 19, 39	1.3/1.7	4.6(−52, −41,5)/5.3(49, −38,1)
Fusiform gyrus	19, 18, 37	1.3/1.1	4.5(−39, −73, −10)/3.6(27, −84, −12)
Postcentral gyrus	3, 5	0.6/1.1	3.2(−27, −39,66)/4.1(33, −31,69)

*Angle source 2*:			
Postcentral gyrus	3, 1, 2, 43, 40, 5, 7	11.0/21.2	9.9(−53, −17,60)/15.5(55, −15,55)
Precentral gyrus	6, 4, 43, 44, 9	8.0/22.5	5.9(−53, −7,56)/12.3(52, −10,57)
Inferior and middle frontal gyri	9, 45, 44, 46, 13, 6, 8	6.3/23.1	7.4(−45,24,17)/8.2(58,4, 23)
Inferior and superior parietal lobules	40, 7	5.8/12.9	6.1(−53, −31,43)/5.4(49, −39,39)
Middle and inferior occipital gyri	18, 37, 19	3.3/1.3	6.0(−27, −103,7)/4.8(39, −68,2)
Insula	13	0.2/0.4	4.9(−39,24,17)/5.9(40,23,17)
Supramarginal gyrus	40	0.9/2.8	4.6(−48, −49,31)/5.9(46, −43,37)
Cuneus	18, 19	2.4/na	5.7(−16, −104,11)/na
Posterior cingulate	23, 30, 29	2.2/0.9	5.6(0, −38,23)/4.7(4, −35,25)
Inferior and superior temporal gyri	19, 20, 37, 22, 42, 39, 41	3.5/4.3	5.1(−48, −63, −4)/4.7(40, −66, −3)
Fusiform gyrus	37, 19, 20, 18	1.7/1.1	4.9(−40, −54, −7)/4.1(42, −62, −7)
Superior frontal gyrus	8, 11	na/1.7	na/4.4(40,18,47)
Orbital gyrus	11, 47	0.9/1.9	3.5(−16,23, −29)/4.0(7,45, −30)

*Angle source 3*:			
Cuneus	19, 18, 7	13.4/11.9	16.9(−19, −90, 39)/13.2(16, − 90, 36)
Precuneus	19, 7, 31, 39	16.2/14.9	16.2(−21, −85, 43)/15.4(16, −88, 42)
Superior parietal lobule	7, 5	11.2/10.2	11.7(−19, − 69, 57)/9.0(15, − 70, 59)
Middle and superior occipital gyri	18, 19	6.5/3.3	10.5(−21, −98, 24)/7.1(27, −96, 24)
Inferior and superior temporal gyri	20, 22, 13, 39	5.4/2.2	8.1(−50, − 23, − 33)/5.7(52, − 26, − 31)
Superior frontal gyrus	11, 10	2.8/1.5	8.0(−7, 58, − 27)/4.4(22, 70, − 1)
Rectal gyrus	11	1.7/0.9	7.2(−1, 23, − 29)/6.2(4, 25, − 29)
Fusiform gyrus	20	2.6/1.5	6.3(−59, − 19, − 29)⁄5.8(58, − 26, − 30)
Postcentral gyrus	7, 3, 5, 2, 4	5.8/1.5	5.4(−15, − 55, 65)/4.3(4, − 40, 66)
Inferior parietal lobule	7, 39, 40	2.2/na	4.9(−39, −66,45)/na

*Angle source 4*:			
Precentral gyrus	6, 4, 44, 9	20.7/6.0	19.3(−39, −7,61)/5.4(36, −9,57)
Middle and superior frontal gyri	6, 8, 9, 11, 46, 10	26.4/6.5	15.7(−33, −7,61)/7.9(28,24,38)
Inferior parietal lobule	40	7.1/4.1	11.1(−39, −34,39)/8.4(33, − 39,39)
Postcentral gyrus	3, 5, 2, 1	8.0/0.4	10.2(−48, −19,64)/3.1(48, − 30,35)
Insula	13	0.4/2.6	5.6(−34,21,9)/6.7(40,18,9)
Inferior and medial frontal gyri	13, 44, 45, 9, 47, 6, 25	4.8/3.6	6.7(−40,21,7)/6.0(45,15,12)
Precuneus	7, 31, 19	1.7/0.4	4.9(−22, −81,50)/5.9(30, −43,42)
Inferior semi-lunar lobule		3.0/na	5.9(−45, −71, −46)/na
Cerebellar tonsil		1.7/na	5.6(−48, −65, −46)/na
Superior parietal lobule	7	1.3/na	5.2(−27, −69,46)/na
cuneus	18, 17	1.1/1.1	4.5(−1, −103,4)/4.6(1, −103,9)

*Angle source 5*:			
Middle and superior frontal gyri	9, 10, 8, 46, 6, 11	39.6/46.9	10.0(−39,25,32)/11.5(34,33,25)
Precentral gyrus	9	0.6/1.3	9.0(−39,21,36)/11.1(36,22,35)
Cuneus	17, 18, 23, 30, 19	1.9/9.3	4.1(−13, −93,35)/7.3(18, −72,9)
Medial and inferior frontal gyri	9, 8, 11, 6, 47, 48	5.0/7.6	4.5(−1,47,42)/6.2(9,50,36)
Rectal gyrus	11	1.5/1.5	5.0(−3,29, −29)/5.9(3,29, −28)
Lingual gyrus	18, 17	0.2/2.2	3.5(−25, −59,8)/5.7(7, −102, −9)
Inferior parietal lobule	40	2.2/na	5.6(−50, −39,28)/na
Orbital gyrus	11	0.6/1.9	4.0(−3,35, −31)/4.9(9,51, −28)
Inferior temporal gyrus	20, 21	1.1/0.4	4.1(−61, −23, −17)/3.3(50, −11, −20)

*Angle source 6*:			
Lingual gyrus	17, 18, 19	10.2/0.9	12.8(−15, −86, −2)/4.6(30, −73, −4)
Middle and inferior frontal gyri	9, 46, 10, 11, 6, 8, 47, 45	8.4/10.1	10.6(−36,13,28)/11.6(34,14,27)
Orbital gyrus	11, 47	0.2/4.1	3.1(−18,25, −23)/10.2(15,37, −22)
Cuneus	17, 18, 19, 30, 23, 7	13.8/1.5	9.6(−12, −91,6)/4.3(16, −71,9)
Parahippocampal gyrus	36, 35, 28, Hippocampus, 30, Amygdala	4.3/2.4	9.3(−25, −20, −28)/7.0(21, −22, −29)
Precentral gyrus	9, 6, 4	1.7/2.6	7.5(−33, 9, 31)/9.1(36,13, 32)
Rectal gyrus	11	na/1.9	na/8.1(10, 40, − 20)
Middle occipital gyrus	18, 19	3.7/0.9	8.0(−12, − 90,13)/5.6(33, − 76,15)
Middle and superior temporal gyri	39, 19, 21, 22, 37, 13, 41, 42	3.9/4.1	7.8(−34, −72,17)/6.0(56, −39,13)
Superior and medial frontal gyri	10, 11, 6, 25, 9	2.4/4.8	7.5(−27, 48, 2)/5.9 (24, 48, 3)
Cerebellar tonsil		16.4/6.7	7.5(−28, − 47, − 37)/6.4 (25, − 38, − 31)
Cerebellar vermis		3.2/3.0	7.3(−21, − 18, − 31)/7.6 (24, − 26, − 31)
Insula	13	0.9/1.1	7.3(−37,18,18)/4.7(50, −39,13)
Cingulate gyrus	24, 31	1.7/na	5.7(−10, −2,39)/na
Precuneus	31, 7, 19	0.9/1.1	4.1(−25, −62,34)/4.5(28, −76,19)
Posterior cingulate	30	1.1/0.6	4.5(−28, −71,16)/3.8(18, −65,7)
Postcentral gyrus	3, 5, 1, 2	1.7/1.1	4.4(−42, −23,67)/3.6(71, −16,26)
Inferior parietal lobule	40	1.5/0.2	4.2(−42, − 39, 38)/3.2(52, −48,54)
Thalamus		1.3/na	4.1(−16, −27,7)/na

*Power source*:			
Insula	13, 40, 41, 22	8.2/10.8	11.0(−45,7, 0)/13.8(43,10, −7)
Superior and transverse temporal gyri	22, 38, 13, 41, 42	17.0/15.7	13.8(−46,7, −5)/13.2(45,4, −5)
Inferior and medial frontal gyri	47, 13, 45, 9, 11, 10, 6, 25, 8	20.1/12.1	12.6(−46,13, −4)/12.2(42,16, −8)
Precentral and postcentral gyri	6, 13, 44, 43, 40	5.6/2.4	12.2(−46, −7,6)/5.5(53, −6,6)
Anterior cingulate and cingulate gyrus	32, 24, 25, 10	11.0/3.9	9.0(−1,42,2)/6.1(4,44,3)
Claustrum		0.9/1.7	5.2(−37, −10,7)/5.8(37, −13,8)
Parahippocampal gyrus	30, 35, 34, 27, 28, 36, Amygdala	3.5/3.9	5.4(−15, −32, −5)/5.4(13, −34, −3)
Inferior parietal lobule	40	1.1/0.2	5.0(−61, −24,23)/3.1(55, −31,22)
Thalamus		1.9/0.6	4.7(0, −17,6)/3.2(15, −33,2)
Cerebellar vermis		2.8/3.2	4.4(−21, −34, −13)/5.9(24,5, −22)

**Table 4:** White matter labels for networks detected by SBM. Voxels above a threshold of |*Z*| > 3.0 were converted from Montreal Neurological Institute (MNI) coordinates to the ICBM DTI-81 coordinates and entered into a database to assign anatomic labels. The volume of significant white matter voxels within each fiber tract area is provided in cubic centimeters (cc). The areas with volume above 0.1 are listed. The percentage of the fiber tract containing significant white matter voxels is also provided. Within each fiber tract, the maximum *Z* value and its coordinate are provided.

Angle source 1	L/R volume (cc)	L/R percentage (%)	L/R max⁡ *Z*(*x*, *y*, *z*)
Fornix (column and body of fornix)	0.38	62.64	7.15(−3, −4.5,13.5)
Retrolenticular part of internal capsule	na/0.73	na/19.04	na/6.71(−10.5, −4.5,6)
Superior cerebellar peduncle	0.18/na	19.22/na	6.52(6, −33, −12)/na
Cingulum (hippocampus)	0.114/0.44	9.71/34.76	4.25(−18, −31.5, −7.5)/6.32(18, −33, −3)
Cerebral peduncle	0.29/0.10	12.83/10.25	6.31(12, −25.5, −6)/5.11(−6, −33, −12)
Fornix (cres)/Stria terminalis	0.003/0.17	0.30/16.24	3.16(−21, −31.5,12)/6.28(19.5, −30,12)
Posterior limb of internal capsule	0.49/0.02	12.90/0.55	5.97(16.5, −15,9)/3.73(−10.5,0, 7.5)
Anterior limb of internal capsule	na/0.22	na/9.55	na/5.01(−13.5, −25.5, −6)

b	L/R volume (cc)	L/R percentage (%)	L/R max *Z*(*x*, *y*, *z*)

Splenium of corpus callosum	1.75	13.70	5.41(0, −39,22.5)

Angle source 4	L/R volume (cc)	L/R percentage (%)	L/R max *Z*(*x*, *y*, *z*)

Superior longitudinal fasciculus	0.47/0.21	7.21/3.20	7.68(−39, −33,37.5)/5.61(31.5, −40.5,36)

Angle source 5	L/R volume (cc)	L/R percentage (%)	L/R max *Z*(*x*, *y*, *z*)

Middle cerebellar peduncle	0.39	2.48	3.87(−24, −61.5, −30)

Angle source 6	L/R volume (cc)	L/R percentage (%)	L/R max *Z*(*x*, *y*, *z*)

Superior longitudinal fasciculus	0.29/0.49	4.43/7.31	8.89(−34.5,12,25.5)/8.60(31.5,9, 28.5)
Middle cerebellar peduncle	1.18	7.52	5.74(25.5, −40.5, −37.5)
Cingulum (cingulate gyrus)	0.56/na	21.15/na	5.61(−10.5, −4.5,40.5)/na
Cingulum (hippocampus)	0.13/na	11.47/na	5.10(−27, −19.5, −24)/na
Superior corona radiata	0.12/0.02	1.63/0.32	4.67(−28.5,12,28.5)/3.63(27,7.5,28.5)

Power source	L/R volume (cc)	L/R percentage (%)	L/R max *Z*(*x*, *y*, *z*)

Cingulum (hippocampus)	0.46/0.36	40/28.6	5.08(−18, −31.5, −9)/4.91(18, −30, −10.5)
Uncinate fasciculus	0.04/0.08	10/22.12	4.30(−39,0, −15)/5.22(36,3, −15)

**Table 5:** Comparison of results with previous SBM analysis.

Region	SBM of gray matter [22]	SBM of angle	SBM of power
Bilateral temporal lobe	X		X
Thalamus	X	X	
Basal ganglia	X		
Frontal 1	X	X	
Parietal	X	X	
Frontal 2		X	
Right precentral		X	
Left precentral		X	
